# RFX1 and RFX3 Transcription Factors Interact with the D Sequence of Adeno-Associated Virus Inverted Terminal Repeat and Regulate AAV Transduction

**DOI:** 10.1038/s41598-017-18604-3

**Published:** 2018-01-09

**Authors:** Laura Julien, Julie Chassagne, Cécile Peccate, Stéphanie Lorain, France Piétri-Rouxel, Olivier Danos, Sofia Benkhelifa-Ziyyat

**Affiliations:** 1 Sorbonne Universités UPMC Univ Paris 06, Inserm, Institut de Myologie, Centre de Recherche en Myologie (CRM), GH Pitié Salpêtrière, 105 bd de l’Hôpital, Paris, 13 France; 2 REGENXBIO, 9600 Blackwell Rd, Rockville, MD 20850 USA

## Abstract

Adeno-associated virus (AAV) transduction efficiency depends on the way in which cellular proteins process viral genomes in the nucleus. In this study, we have investigated the binding of nuclear proteins to the double stranded D (dsD) sequence of the AAV inverted terminal repeat (ITRs) by electromobility shift assay. We present here several lines of evidence that transcription factors belonging to the RFX protein family bind specifically and selectively to AAV2 and AAV1 dsD sequences. Using supershift experiments, we characterize complexes containing RFX1 homodimers and RFX1/RFX3 heterodimers. Following transduction of HEK-293 cells, the AAV genome can be pulled-down by RFX1 and RFX3 antibodies. Moreover, our data suggest that RFX proteins which interact with transcriptional enhancers of several mammalian DNA viruses, can act as regulators of AAV mediated transgene expression.

## Introduction

Adeno-associated virus (AAV) is a helper-dependent parvovirus with a small single-stranded (ss) DNA genome. Its replication depends on the activity of cellular factors which are triggered by co-infecting helper viruses or, albeit with a lower efficiency, by a variety of genotoxic stresses^1,2^. These factors are involved in every step of the viral life cycle, including the conversion of the ssDNA into a double-stranded (ds) transcription template, the production of viral transcripts, and the assembly of capsids. A number of cellular proteins interact with the palindromic Inverted Terminal Repeat (ITR), a 145 nucleotides element which folds into a T-shaped duplex structure (A′-B′-B-C′-C-A) at both extremities of the viral genome^2,3^. ITRs are required for replication, packaging, integration into the host cell DNA and proviral rescue, and they also contain promoter elements^4,5^. This short sequence is the only viral information present in gene transfer vectors derived from AAV. Recombinant AAV vectors (rAAV) are remarkably efficient for the transduction of certain cells and tissues, especially in the neuromuscular system, whereas some cellular targets appear to be much less permissive^6–8^. Although receptor attachment determines AAV vector tropism, several steps after internalisation including viral trafficking, nuclear entry, uncoating, and second-strand synthesis pose significant barriers to functional transduction^8^. Cell permissivity and transduction efficiency also relie on still poorly characterized interactions of cellular factors with the ITRs. Proteins active in double strand break repair recognize the ITRs and regulate the conversion of ssDNA into transcriptionally active dsDNA^9,10^. They are also involved in the formation of large concatemers of vector genomes^11–14^ and in the stabilization of transcriptionally active transgene expression cassettes^15^. FKBP52, a steroid receptor-associated immunophilin, also binds the ITR in a phosphorylation dependent manner and has been associated with cell permissivity to AAV vectors^16–18^. Multiple transcription factors binding sites are present in the ITR sequence^19,20^ and binding has been documented for the C-AMP response element-binding protein (CREB) and the NF-κB-repressing factor (NRF)^19,21^.

In this study, we have further investigated the nature of nuclear proteins able to bind the double stranded D sequence of the AAV2 ITR (D2) using electromobility shift assays (EMSA). We report that transcription factors from the RFX family bind the D2 sequence and regulate AAV transduction. RFX (Regulatory factor X) proteins are transcription factors with a highly conserved DNA-binding domain that recognizes the X-box motif in several promoters^22–24^. Eight mammalian RFX factors (RFX1-8) have been identified, RFX1-3 being the most studied^25^. RFX1, RFX2, and RFX3 bind their target sites as monomers, homodimers and heterodimers and are involved in the regulation of viral and cellular genes^26–33^. Here, we demonstrate that RFX1 homodimer and RFX1/RFX3 heterodimer form specific complexes with D2. We also showed that RFX1 and RFX3 interact specifically with D2 in rAAV transduced HEK-293 cells and modulate AAV mediated transgene expression.

## Results

### Nuclear factors bind the double stranded D2 of AAV ITR

**Figure 1:**
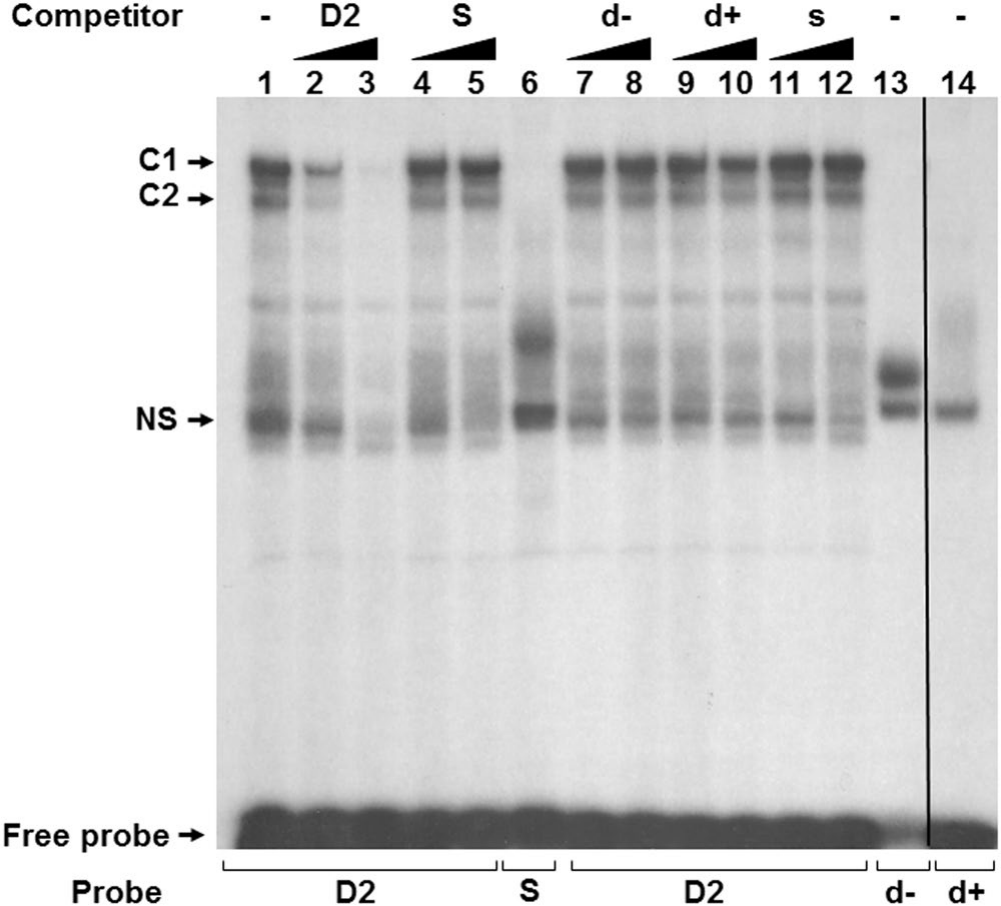
Binding of nuclear factors to the double stranded (ds) D sequence of AAV2 (D2) analysed by EMSA using HEK-293 cells nuclear extracts. The binding reaction was performed in absence of competitor DNA (lane 1) or in presence of increasing concentration of ds competitors, D2 (lanes 2 and 3) or S (lane 4 and 5) or unlabeled ssDNA of these sequences (d−, d+ and s) (lanes 7 to 12). The binding reaction was also performes with d− and d+ probes (lanes 13 and 14). C1 and C2 complexes were formed specifically with D2. Unknown shifted complexes were observed with S, d− and d+ probes (lanes 6, 13 and 14). NS indicate non-specific protein(s) binding to the oligonucleotides used.

To analyse cellular factors able to bind the double stranded (ds) sequence from AAV2 (D2), we performed electrophoretic mobility shift assays (EMSA) with nuclear extracts (NE) from HEK-293 cells. As shown in Fig. 1, a D2 probe promoted the formation of two complexes, designated C1 (major) and C2 (minor) respectively (Fig. 1, lane 1). To verify the binding specificity of C1 and C2 complexes, HEK-293 NE were incubated with the D2 probe in the presence of 100-fold or 200-fold molar excess of unlabeled ds competitor oligonucleotides. The D2 sequence was able to compete efficiently for C1 and C2 complexes formation (lanes 2 and 3), whereas an excess of the unrelated ds sequence (S) was unable to do so (lanes 4 and 5). Competition experiments with these sequences as single stranded (ss) DNA (d−, s) (lanes 7-8 and 11–12) had no effect on complex formation. While d+ inhibit slithly D2 binding in competition experiments (lanes 9 and 10), no detectable C1 and C2 complexes were observed with d+ (lane 14), d− (lane 13) and S (lane 6). D2 binding was also observed using nuclear extracts from HeLa and SUP-T1 cells (Supplementary Fig. S1). Thus cellular factors present in the HEK-293 NE are able to bind specifically the ds D sequence of AAV2.

### Analysis of C1 and C2 complex formation on D sequences of other AAV serotypes

**Figure 2:**
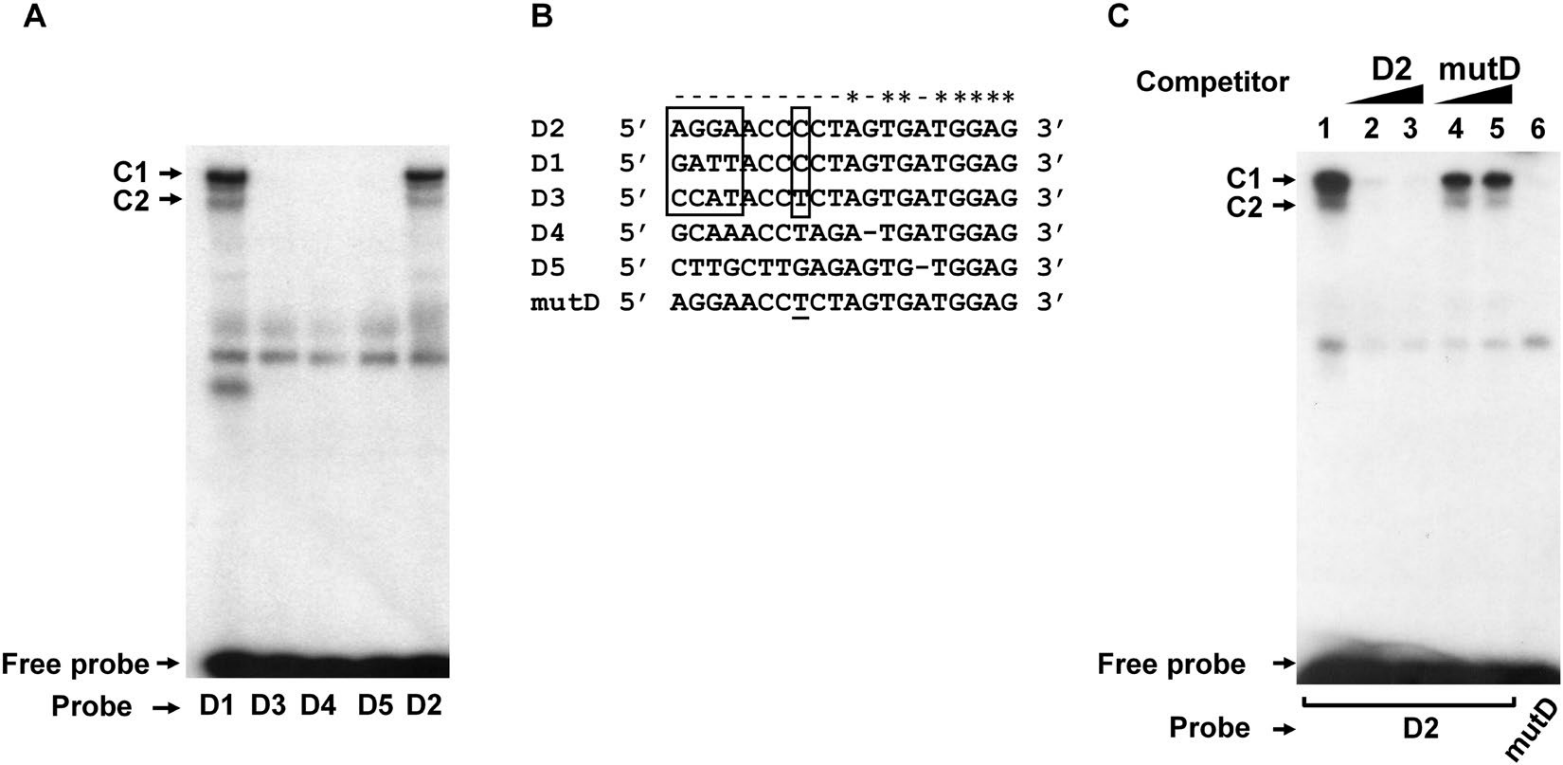
(**A**) Analysis of C1 and C2 complex formation on D sequences of AAV serotypes. 293 cells NE was used in EMSA with dsD sequences from AAV1 to AAV5 (D1 to D5). (**B**) Sequence alignment of D sequences of AAV serotypes. The variable sequence in D1, D2 and D3 is delineated by boxes. mutD represent the mutated D2 sequence. (**C**) Identification of nucleotide requirement of C1 and C2 complexes binding to the D2 sequence. EMSA was carried out with the D2 sequence in absence of competitor DNA (lane 1) or in presence of 200-fold molar excess of unlabeled D2 (lanes 2) or mutD (lane 3) competitors. mutD oligonucleotide was also used as a probe (lane 4). The Black line delineate the boundary between not contiguous lanes of the same gel. (_*_) Identical nucleotides and (−) differents nucleotides in D1–D5 sequences.

The formation of complexes was also studied using D sequences from AAV serotypes 1 to 5 (D1 to D5). Figure 2A shows that C1 and C2 complexes were only detected with D1 and D2 and that the relative quantities of these two complexes were conserved. D1 and D2 sequences display differences in the first four nucleotides (boxed in Fig. 2B) suggesting that those are not necessary for C1 and C2 complex formation. Interestingly, the D3 sequence, which did not form complexes (Fig. 2A, lane 2), displays one additional change at position 8 (boxed in Fig. 2B). This nucleotide is also different in the more divergent D4 and D5 sequences which also failed to form complexes. To verify whether this nucleotide is important for C1 and C2 complex formation, a C to T substitution was introduced in D2 and the mutated D2 oligonucleotide (mutD in Fig. 2B) was analysed for its ability to form complexes (Fig. 2C). mutD was unable to compete with D2 (lanes 3) and was unable to form the C1 and C2 complexes (lane 4) indicating that C1 and C2 complex formation requires a C nucleotide at position 8 in the D sequence of AAV.

### RFX proteins bind specifically to the D2 sequence

**Figure 3:**
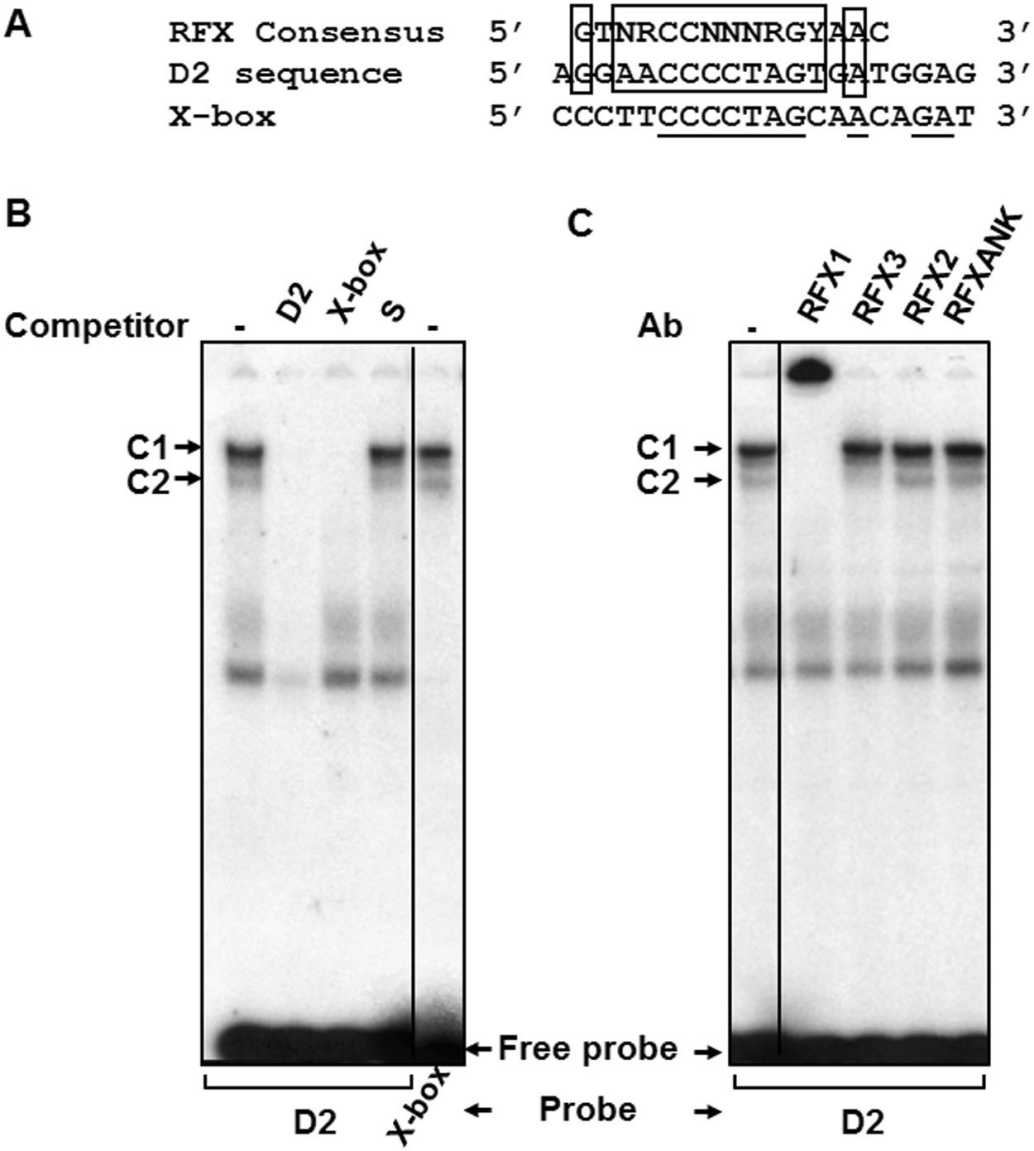
Specific binding of RFX proteins to the D2 sequence in HEK-293 NE. (**A**) Alignment of D2 sequence with the RFX binding consensus sequence and the MHC class II X-box site in the HLA-DRA (X-box). Boxes indicate positions that match the consensus sequence and identities between the X-box and the D2 sequence are underlined. R, Y and N represent a purine, a pyrimidine, and any nucleotide, respectively. (**B**) EMSA was performed with the D2 sequence in absence of competitor DNA (lane 1), in the presence of the unlabeled D2 (lane 2), unlabeled X-box (lane 3) or unlabeled S oligonucleotides (lane 4). X-box was also used as a probe (lane 5). (**C**) C1 and C2 complexes were formed by using the D2 probe and HEK-293 NE in EMSA. Antibodies against human RFX1, RFX2, RFX3 and RFXANK were added to the reaction as described in the text (n = 3 experiments). Black lines delineate the boundary between not contiguous lanes of the same gel.

The DNA sequence alignment shown in Fig. 3A reveals homologies between the D2 sequence, the consensus binding motif for the RFX family of transcription factors and the X-DRA sequence (X-box). The X-box found in the promoter of HLA class II genes is efficiently recognized by all members of the RFX family including RFX1, RFX2, RFX3 and RFX5^28,34^. RFX5 has a different structure and associates with the HLA class II promoter in combination with two other factors, RFXAP and RFXANK/RFXB^35^. To test the possibility that proteins related to the RFX family form complexes with the D sequence, a radiolabeled D2 probe was incubated with HEK-293 NE in the presence of unlabeled X-box. Figure 3B shows that X-box was competing for complexes formation (lane 3) as efficiently as D2 itself (lane 2). Additionnaly, complexes with the same mobility as C1 and C2 were obtained using X-box as a probe (lane 5) suggesting that common proteins bind to both motifs.

The presence of RFX proteins in C1 and C2 was assessed using antisera against human RFX1, RFX2, RFX3 or RFXANK. Figure 3C indicates that the RFX1 antiserum supershifted both C1 and C2 complexes. The RFX3 antiserum supershifted the C2 complex but did not affect C1. In contrast, C1 and C2 complexes mobility was not affected by RFX2 or RFXANK antisera. These findings indicate that both complexes contain the RFX1 protein and that the C2 complex contains also RFX3.

### *In vitro* synthesized RFX1 and RFX3 bind specifically to the D2 sequence

**Figure 4:**
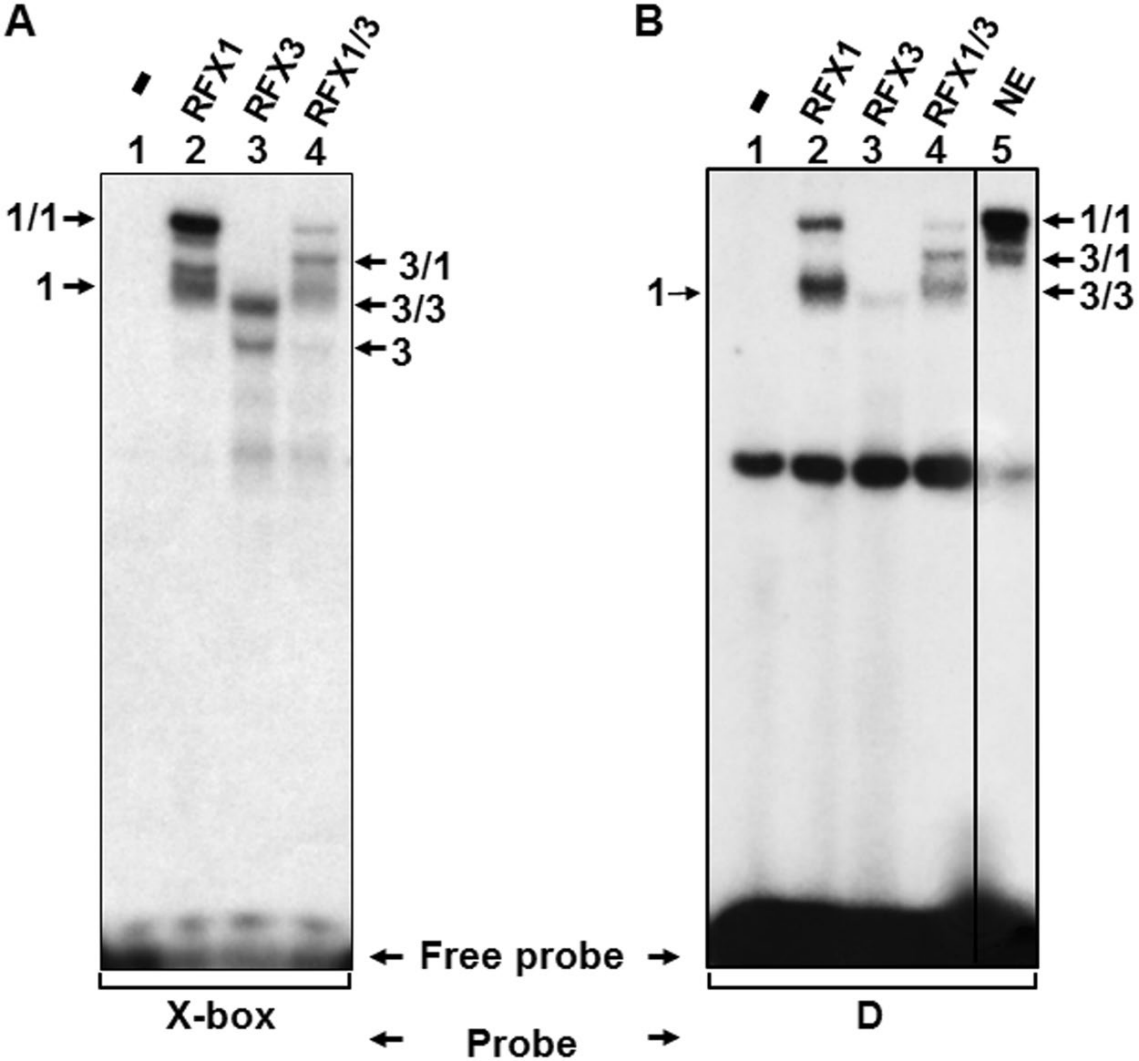
Binding of *in vitro* synthesized RFX1 and RFX3 to the D2 sequence. (**A**) Binding reaction was preformed with a X-box oligonucleotide in the presence of human RFX1 (lane 2) or RFX3 (lane 3) individually synthesized *in vitro* or the presence of cosynthesized RFX1 and RFX3 (lane 4). A control translation product (pSG5 vector alone) was incubated with the X-box probe (lane1). Complexes formed by RFX1 or RFX3 monomers (1 or 3), RFX1 or RFX3 homodimers (1/1 or 3/3) or RFX1/RFX3 heterodimers (3/1) are indicated. (**B**) Same experiment as for panel A with the D2 oligonucleotide. For comparison C1 and C2 complexes formed with HEK-293 NE and the D2 oligonucleotide were loaded in lane 5. The Black line delineate the boundary between not contiguous lanes of the same gel.

To confirm the hypothesis that RFX1 and RFX3 bind to the D2 sequence, we examined the ability of D2 and X-box to bind mono- and homodimer of RFX1 or RFX3 as well as the RFX1/RFX3 heterodimer using *in vitro* translated proteins. Homogeneity of *in vitro* translated protein was verified by parallel transcription/translation reactions in presence of 35S-labeled methionine (Supplementary Fig. S2). As previously reported^28^, RFX1 or RFX3, translated alone, generated distinguishable patterns of retarded bands with X-box (Fig. 4A, lane 2 and 3 respectively) corresponding to monomeric (1 or 3) and homodimeric (1/1 or 3/3) forms of RFX1 or RFX3. Cotranslation products generated an additional complex with an intermediate electrophoretic mobility, corresponding to RFX1/RFX3 heterodimers (Fig. 4A, lane 4). When the D2 sequence was used as a probe, only a weak binding activity was observed for monomeric and dimeric RFX3 (Fig. 4B, lane 3). In contrast, complexes were readily formed with RFX1 monomers and homodimers, as well as with RFX1/RFX3 heterodimers (Fig. 4B, lane 2 and 4). These complexes comigrated with the C1 and C2 complexes detected using HEK-293 NE (lane 5). We therefore concluded that the major C1 complex corresponds to an RFX1 homodimer and that the minor complex C2 corresponds to an RFX1/RFX3 heterodimer.

### RFX1 downregulation impacts AAV mediated transgene expression

**Figure 5:**
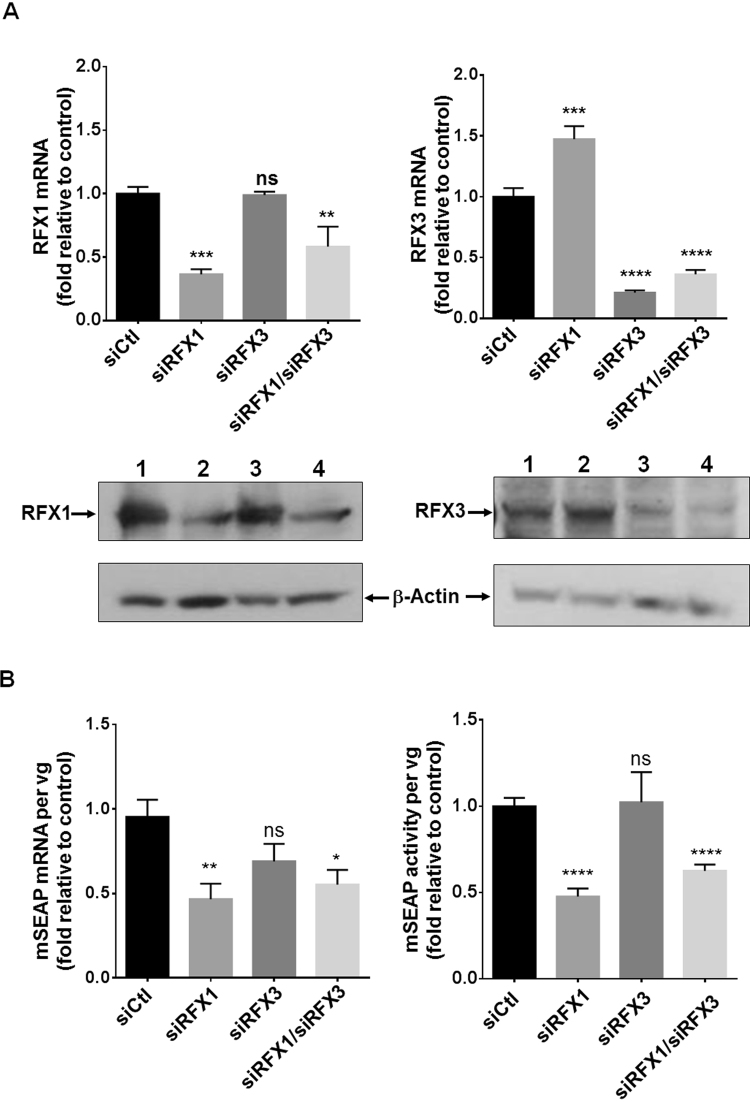
siRNA-mediated knockdown of RFX proteins impacts AAV mediated transgene expression in HEK-293 cells. (**A**) Real time RT-PCR and western bloting analysis of RFX expression showing the efficacy of RFX siRNA silencing in HEK-293 cells transfected with control (lane1) siRNA (siCtl), siRNAs against either (lane2) RFX1, (lane 3) RFX3 or (lane4) both (respectively siRFX1, siRFX3, siRFX1/RFX3) (n = 3 independent experiments). (**B**) siRNA-mediated knockdown of RFX proteins impacts AAV transduction in HEK-293 cells. siRFX1, siRFX3 and siRFX1/RFX3 transfected cells were transduced with an AAV encoding the murine secreted alkaline phosphatase reporter gene (AAV-mSEAP) 24 h post-transfection (n = 4 independent experiments). The AAV-mSEAP transduction efficiency was assessed in each condition by quantifying the mSEAP activity in cell supernatants and cellular mSEAP mRNA expression levels 24 h post-transduction. mSEAP activity and transcripts levels were normalized by the AAV vg copy number. The data are represented as the mean ± SEM of at least three independent experiments. Data that were statistically different from siCtl are marked *p < 0.05; **p < 0.01, ***p < 0.001, ****p < 0.0001. ns, non-significant.

To explore the possible involvement of RFX1 and RFX3 in AAV transduction efficiency, HEK-293 cells transfected with siRNAs against either RFX1, RFX3 (respectively siRFX1, siRFX3) or both (siRFX1/RFX3) as well as control (siCtl) (Fig. 5A) were transduced with an AAV vector encoding the murine secreted alkaline phosphatase (AAV-mSEAP) reporter gene. The transduction efficiency was assessed by quantifying the mSEAP activity in cell supernatants and mSEAP mRNA expression levels. RFX1 mRNA levels were decreased in siRFX1 and siRFX1/RFX3 transfected cells by 64% and 42% respectively and unmodified in siRFX3 transfected cells (Fig. 5A). RFX3 mRNA was specifically reduced by 79% and 64% respectively in siRFX3 and siRFX1/RFX3 transfected cells compared to siCtl transfected cells. Of note, in siRFX1 transfected cells, the RFX3 mRNA level was upregulated by 47% compared to siCtl cells, showing that RFX1 could negatively regulate RFX3 transcription. In accordance with the RT-PCR results, western blotting showed that RFX1 and RFX3 proteins levels were reduced respectively in siRFX1 and siRFX3 transfected cells and that RFX3 was upregulated in siRFX1 treated cells. Accordingly, it has previously shown that RFX1 represses the activity of RFX3 promoter as well as its own promoter^36,37^. RFX1 knock down was associated with reduced AAV transgene expression following cell transduction. Compared to control, mSEAP mRNA levels and mSEAP activity were decreased by 54% and 38% respectively indicating that RFX1 plays a role in AAV mediated transgene expression (Fig. 5B). In siRFX3 transfected cells, no statistical difference of mSEAP expression levels was observed between siRFX3 and siCtl transfected cells (Fig. 5B) suggesting that, unlike RFX1, RFX3 does not affect AAV transgene expression. Thus, the AAV expression defect observed in siRFX1/RFX3 transfected cells seems to be exclusively due to RFX1 downregulation.

### A single mutation in D2 sequence influences AAV transduction efficiency

**Figure 6:**
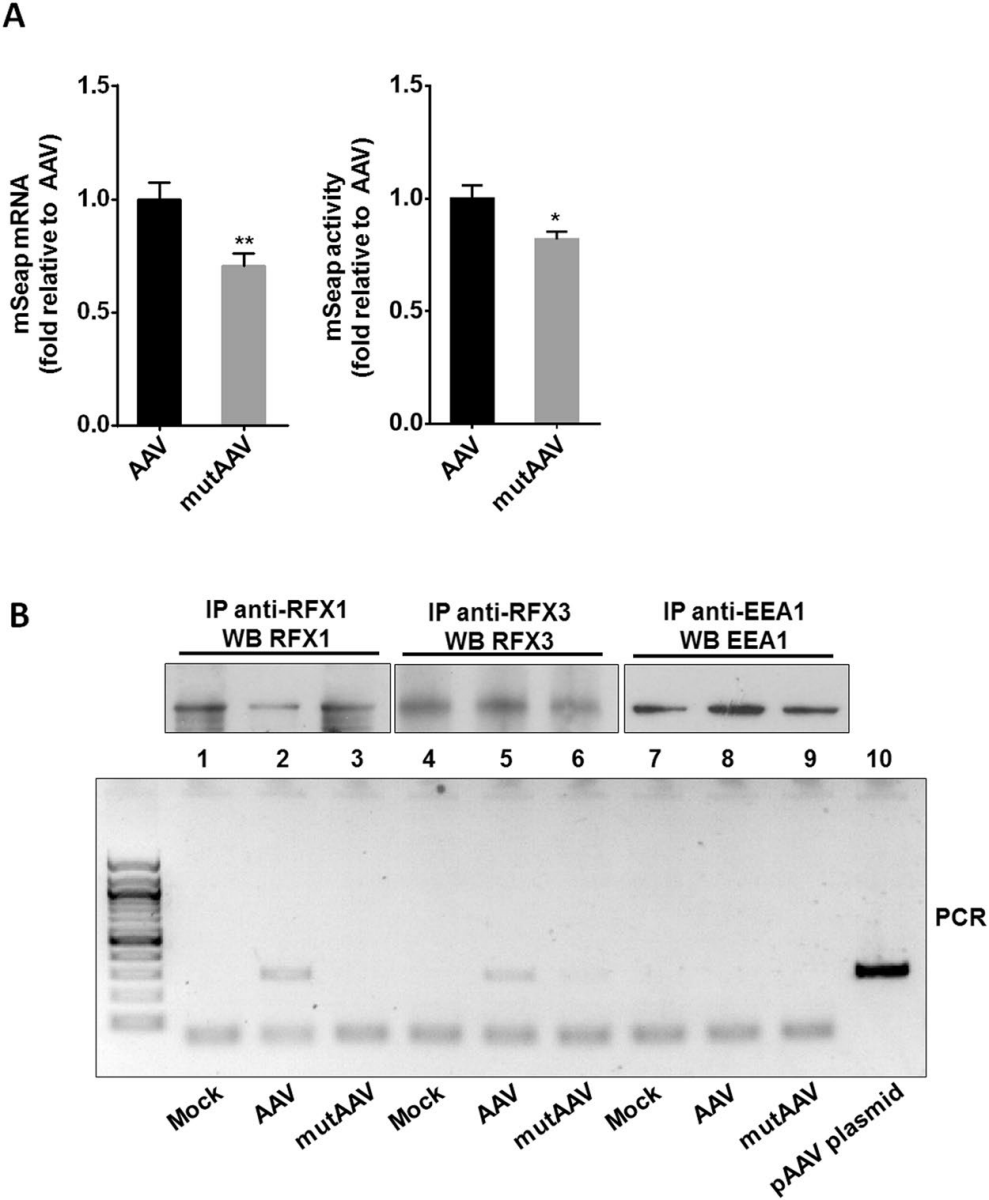
Mutation in D2 sequence impacts AAV transduction efficiency and RFX proteins binding. (**A**) Transduction efficiency of an AAV vector carrying the mutated D sequence (mutAAV-mSEAP) and the unmodified vector (AAV-mSEAP) in HEK-293 cells. Transduction was assessed 24 h post-transduction in each condition, by quantifying the mSEAP activity in cell supernatants and cellular mSEAP mRNA expression levels that were normalized by the AAV vg copy number. The data are represented as the mean ± SEM of three independent experiments using three different batches of AAV-mSEAP and mutAAV-mSEAP (*p < 0.05; **p < 0.01). **(B)** Detection of AAV DNA in RFX1 and RFX3 immunoprecipitates. HEK-293 cells were mock transduced (Mock) or transduced with either AAV or mutAAV and analysed 24 h post-transduction. RFX1 and RFX3 immunoprecipitation from cell extracts were analysed by western blotting with anti-RFX1 and anti-RFX3 antibodies (Rabbit IgG). As expected RFX proteins were present in immunoprecipitates of mock transduced or transduced cells with either AAV or mutAAV vectors (lanes 1–6). AAV DNA was detected using PCR on the pulled down material. Negative PCR controls included immunoprecipitations with an anti-EEA1 antibody (rabbit IgG) which does not precipitates RFX proteins (lane 7, 8 and 9) as well as with Protein A beads alone (not shown). The pAAV plasmid was used as positive control (lane 10).

To evaluate whether the C to T substitution in the D2 sequence affects viral transduction, a pseudotyped AAV1/2 vector carrying the mutated D2 sequence and encoding the mSEAP (mutAAV-mSEAP) was produced and titered side by side with the unmodified vector (AAV-mSEAP). mutAAV and AAV showed equal titers indicating that the mutation did not affect vector production (Supplementary Fig. S3). Moreover, AAV genomes analyzed by denaturating agarose gel electrophoresis showed that the single-stranded full-length DNAs of either mutAAV-mSEAP or AAV-mSEAP are equally packaged in particles (Supplementary S3). HEK-293 T cells were transduced and analysed for transgene expression by quantifying mSEAP activity in cell supernatants. Consistent with our siRNA experiments, our results showed a slight but significant decrease of mSEAP transcripts (30%) and activity levels (20%) (Fig. 6A). It is noteworthy that mutAAV-mSEAP expression was not affected by the knock down of RFX1 and RFX3 (Supplementary Fig. S4). Our results showed that AAV vector containing the single point mutation in the D sequence exhibits a slight decreased transduction efficiency.

### Efficient pull down of AAV genome from RFX immunoprecipitations

To confirm the binding of RFX protein to D2 sequence in the viral context, we examined the ability of RFX proteins to bind the AAV vector genome in pull down experiments. HEK-293 cells were mock-transduced or transduced with either AAV or mutAAV vectors. Whole cell extracts were prepared and the presence of either RFX proteins or AAV genomes were analyzed respectively by western blotting (WB) and by PCR on the pulled down material from same anti-RFX1 and -RFX3 immunoprecipitations samples. As expected, WB showed that RFX1 and RFX3 proteins were readily detected in mock, AAV and mutAAV transduced cells (Fig. 6B, lanes 1–6). PCR analysis on the pulled down material from anti-RFX proteins shown in Fig. 6B indicated that the AAV viral DNA was associated with RFX1 and RFX3 (lanes 2 and 5) while AAVmut genome was not (lanes 3 and 6). Immunoprecipitations with an Early Endosome Antigen 1 antibody (anti-EEA1) which does not precipitate RFX proteins (Fig. 6B lane 7, 8 and 9 and Fig. S5) as well as with protein A beads alone (Fig. S5, negative PCR not shown) were included as a negative PCR control. The pAAV plasmid was used as positive control (lane 10). Altogether, our data indicate that D2 sequence obviously binds RFX proteins in AAV vector context.

## Discussion

There is increasing evidence that, among cellular proteins, transcription factors might be involved in AAV transduction efficiency by interacting with AAV genomes^19,21,38,39^. In this study, we have investigated the nature of the nuclear proteins able to bind the ds D sequence of AAV2 (D2) using HEK-293 nuclear extracts in EMSA. We show, for the first time, that a major C1 complex corresponding to an RFX1 homodimer and a minor one C2, corresponding to an RFX1/RFX3 heterodimer, physically bind D2. These two complexes were also detected in nuclear extracts from HeLa and SUP-T1 cells (Supplementary Fig. S1) consistent with the previous observation that whole cell extracts from HeLa cells contain unidentified protein(s) which bind specifically to D2^40^. We demonstrated that the binding of RFX1 homodimer and RFX1/RFX3 heterodimer to D2 requires a C nucleotide at position 8 which is conserved in D sequences of AAV1 (D1), AAV6, AAV7 and AAV8 but, different in other serotypes as AAV3 (D3), AAV4 (D4) and AAV5 (D5)^41^. Indeed, D1, but not D3, D4 nor D5, have formed RFX complexes in our EMSA experiments. These results suggest a role of RFX proteins in the wild-type AAV life cycle and illustrate the fact that different AAV serotypes may have adapted to different cellular environment not only by selecting different receptors but also by recruiting different cellular factors for the processing of their genome^1,7,20,21,42^.

We observed that AAV-mSEAP transgene expression was decreased in siRFX1 and siRFX1/RFX3 treated HEK-293 cells suggesting a role of RFX1 and RFX1/RFX3 complexes in CMV-driven transgene expression. Indeed, RFX1 and RFX3 both contain a repression and an activation domains which overlap with a dimerization domain and directly regulate transcription upon binding to inverted repeat (called EP or EF-C or MDBP sites) present in the enhancers of several viruses, including hepatitis B virus (HBV) and polyomavirus (Py)^23,28,33,43^. Interestingly, C1 and C2 complexes formed respectively by RFX1 homodimers and RFX1/RFX3 heterodimers are similar to the ones found with the EF-C site in accordance with the role of these proteins in activation of AAV transcription through the D sequence. Indeed, we observed that siRFX1 led to a significant decrease of AAV transgene expression in transduced HEK-293 cells and has no effect on AAV carrying the D2 mutation (mutAAV) (Supplementary Fig. S4) which abolished C1 and C2 formation in EMSA. As previously described^36^, in siRFX1 treated cells, RFX1 knock down increased the RFX3 expression level suggesting a potential regulation of AAV transgene expression by RFX3. Since, no effect was observed in siRFX3 treated cells, RFX1 seems to be the only protein involved in AAV transgene expression. We have demonstrated the binding capacity of RFX proteins to D2 sequence in the context of infectious vector particles. We further showed that RFX1 contained in HEK-293 nuclear extracts and *in vitro* translated RFX proteins displayed identical binding patterns on the D2 and X-box probes in EMSA, suggesting that RFX1 regulates AAV transduction through its binding to D2. It is note worthy that such a binding could be direct or indirect in a physiological context. RFX1 may regulate the promoter activity of the ITR itself^4,5^ or of downstream promoters. For exemple, a zinc finger protein ZF5 that binds the ITR was shown to repress transcription of the AAV p5 promoter^38,39^. More recently, the CREB and NRF transcription factors have been reported to regulate AAV transgene expression through binding to the ITR^20,21,39^. When bound to D2, RFX1 may act alone or in association with these other factors to activate transcription. A similar situation has been described with RFX5 that binds the X-box cooperatively with CREB within the MHC class II gene promoter^44,45^. However, no specific complexes other than RFX1 homodimers and RFX1/RFX3 heterodimers could be identified in the present study. As suggested for CREB^21^, concatemerization and or circularization of the AAV genome could promote the cooperation of RFX1 with other proteins by bringing the distal protein binding sites closer to the D sequence and the promoter region. Alternatively, RFX complexes could compete with other transcription regulators for a common or an overlaping binding site within the D sequence. In this respect, the D sequence was shown to share a NRE-like site^19^ that was previously described as an EP-homologous element regulating the HBV promoter^46^. These hypotheses are supported by our data showing a slight decrease (20%) of transgene expression from mutAAV-mSEAP compared to AAV-mSEAP transduced cells. Indeed, in a physiological context, such a difference in mutAAV may indirectly modify the activity or the binding of other regulator factors on sites surrounding or overlapping D2, leading to a partial compensation of the RFX1 activity on mutAAV transgene expression. In addition, and as suggested for RFX/CREB complex in the context of MHCII promoter, transcription factors binding the ITR may protect AAV promoters against DNA methylation^47,48^.

It will be interesting to determine whether the interaction of RFX1 and RFX3 with the ITR occurs before or after the onset of DNA replication. It is conceivable that the RFX factors bind the D sequence on the incoming genomes, prior to replication. Following particle entry and trafficking through the endosomal pathway, AAV genomes are released from the capsid before they enter the nucleus^42^. These unreplicated ssDNA genomes can adopt two isomeric forms: one with each ITR folded in a T-shaped structure and single-stranded D sequences and the other in which the ITRs, including D, pair into double-stranded DNA. This later structure, called the panhandle, may be recognized by the RFX proteins, stabilized^49^, and may also be more efficiently imported into the nucleus. In this respect, it is worth noting that RFX1 translocates into the nucleus in response to protein kinase C activation^50^. Otherwise, binding of RFX proteins to the D sequence may change DNA conformation and modulate the binding of caretaker proteins and their associated factors^51^ or may directly influence their activity. Indeed, RFX proteins can induce modifications of their partners in multiprotein complexes, as illustrated by the modulation of the auto-phosphorylation capacity of the c-Abl kinase after interaction with RFX1^52^.

Our findings points at RFX1 and RFX3 as ITR-binding cellular factors with a potential influence on the efficacy of AAV-mediated gene transfer. Further studies establishing how these transcription factors regulate transgene expression or genome processing, may guide the design of optimized and better controlled AAV vectors.

## Materials and Methods

### Cells Cultures

HEK-293 cells (ATCC) were maintained at 37 °C and 5% CO2 in Dulbecco’s modified Eagle’s medium (DMEM, Gibco, Life Technologies) that was supplemented with 10% heat-inactivated fetal calf serum, 100 U/ml penicillin, and 100 g/ml streptomycin.

### *In vitro* transcription and translation

Human RFX1 and RFX3 cDNA subcloned respectively into EcoRI and EcoRI-BamHI sites of pSG5 vector (Agilent Technologies) were transcribed and translated *in vitro* using a T7 TNT coupled reticulocyte lysate system (Promega) and used in EMSA with radiolabeled D2 or X-box oligonucleotides.

### Oligonucleotides

Oligonucleotides used in EMSA representing the D sequences of AAV serotypes 1 to 5 (Fig. 2B) or the DRA-X box (X-box) of HLA class II genes (Fig. 3A) or the S sequence (5′-CCAATATTAGATCTGATATCA-3′) were generated by annealing complementary strands and labeling 5′ ends with the polynucleotide kinase and γ-^32^P ATP. Single stranded sequences of D2, d(−) (5′-AGGAACCCCTAGTGATGGAG-3′) and d(+) (5′-CTCCATCACTAGGGGTTCCT-3′), or of S sequences (s) was also used in EMSA as probes or as competitors in experiments whose results are shown in Fig. 1.

### Nuclear extracts and EMSA

Nuclear extracts (NE) were prepared from these HEK-293 cells as previously described^53^. NE or *in vitro* translated products were incubated with 0.2 to 0.5 ng (40,000 cpm) of (γ-^32^P)ATP-labelled oligonucleotides and 2 µg of poly(dI-dC) in 20 µl of 20 mM Hepes (pH 7.9), 60 mM KCl and 4% Ficoll. The binding reaction was carried out for 30 min at 20 °C and then analysed by electrophoresis on 4% polyacrylamide gels (40:2 acrylamide/bisacrylamide) in 0.25 X Tris-Borate-EDTA buffer. For competition experiments, HEK-293 NE were incubated with the D2 or X-box probe in presence of an excess of unlabeled ds or ss oligonuleotides. In supershift experiments binding reactions mixtures were further incubated on ice for 20 mn with the appropriate antiserum before electrophoresis.

### siRNA transfection

HEK-293 cells were seeded at 60,000 per well in 24 well plates. Specific knockdown of RFX1 and RFX3 transcription factors was obtained by a simple or double transfection of siRNA (siRFX1 and siRFX3) (Ambion Silencer Select siRNA, Life technologies) at a final concentration of 6 pmol, using Lipofectamine RNAiMAX (Life technologies) according to the manufacturer’s protocol for adherent cells. In these experiment, Silencer Select Negative Control (Ambion) was used as a control (siCtl).

### AAV transduction and alkaline phosphatase activity quantification

For viral transduction of siRNA treated cells, 24 h after siRNA transfection HEK-293 cells were transduced with AAV1-mSEAP at a multiplicity of infection (MOI) of 5,000 viral genomes per cell. The medium was replaced by fresh medium after 16 hours of AAV incubation. Twenty four hours later, cell supernatants were collected for Alkaline phosphatase activity measurements and cells were harvested for quantifications of mSEAP mRNA and vector genome contents.

Quantification of alkaline phosphatase activity was performed with the Phospha-Light SEAP Reporter Gene Assay System (ThermoFisher) according to manufacturer instructions. Chemiluminescence was measured in microplates in a luminometer (FlexStation 3). Raw data were analyzed through simple spreadsheet and quantified by using standard curve previously prepared by serially diluting stock enzyme.

### Constructs and AAV production

The pAAV-mSEAP construct was mutated in both D sequences (mutpAAV-mSEAP). Briefly, blunt amplicons were obtained by PCR using Phusion polymerase (Thermo Scientific) with the 5′-phosphorylated mutAAV primer ccaactccatcactagAggttccttgtag (with A introducing the T mutation in the D sequence) matching on both D sequences of pAAV-mSEAP plasmid, and cloned in pAAV in MscI restriction sites.

AAV2/1 pseudotyped vectors were prepared by transfection in HEK-293 cells as described previously^54^ using the pAAV-mSEAP or the mutpAAV-mSEAP plasmids with the pXX6 plasmid coding for the Ad helper genes essential for AAV production and the pRepCap plasmid (p0001) coding for AAV1 capsid. Vector particles were purified on iodixanol gradient and concentrated on Amicon Ultra-15 100 K columns (Merck-Millipore). The particle titer corresponding to the number of viral genomes per milliliter (vg/ml) was determined by quantitative real-time PCR on a StepOnePlus (Applied Biosystems), by using the following primers and probe: CTCCATCACTAGGGGTTCCTTG (forward), GTAGATAAGTAGCATGGC (reverse) and TAGTTAATGATTAACCC (Taqman MGB probe, Life Technologies). The pAAV plasmid was used as a control to establish the standard curve for absolute quantification.

### Transcripts and vector genome quantification

Real-time PCRs were performed on a StepOnePlus (Applied Biosystems) using the Power SYBER Green PCR Master mix (Life Technologies) for mSEAP quantification and the ABsolute Qpcr Rox Mix (Life technologies) for vector genome and RFX transcript quantifications.

For vector genome quantification, genomic DNA was extracted from HEK-293 cells using the Puregene Blood kit (Qiagen). Copy number of AAV genomes were measured on 100 ng of genomic DNA using primers described in the previous section. Vector genomes were quantified by using a standard curve prepared by serially diluting the pAAV plasmid.

For transcript quantifications, total RNA was isolated from HEK-293 cells with NucleoSpin® RNA II (Macherey-Nagel), and reverse transcription was performed on 200 ng of RNA by using the Superscript™ II and random primers (Life Technologies). RFX1 and RFX3 transcripts were quantified and normalized to beta-2-microglobulin levels using the following TaqMan Gene Expression Assays (Life Technologies): RFX1 (ID: Hs00172561_m1), RFX3 (ID: Hs00231292_m1) and beta-2-microglobulin (ID: Hs00984230_m1).

mSEAP transcripts were quantified and normalized to the ribosomal phosphoprotein (PO) using the Power SYBER Green PCR Master mix (Life Technologies) and the following primers: mSEAP (forward: 5′CCCTACACTGACTGCGGC3′ and reverse: 5′ATCTGCAGAATTCGCCCTTTC3′) and PO (forward: 5′GGCGACCTGGAAGTCCAACT3′ and reverse: 5′CCATCAGCACCACAGCCTTC3′).

### Imunoprecipitation and PCR on pull-down material

For immunoprecipitations, 2.5 μg of RFX1 or RFX3 antibodies (Novus) were incubated 2 hours with 20 μl of Protein A-coated beads in 100 μl of Ripae buffer and washed three times to remove antibodies excess. 500 μg of cell lysates were incubated 2 hours with 20 μl of Protein A-coated beads pre-bound to the antibodies and the eluted material was analysed by western blotting using the appropriate antibody. For PCR on pull-down experiments, 1/10 of beads of the the RFX1 or RFX3 immunoprecipitations were diluted in 10 μl of Tris/EDTA buffer and subjected to a PCR using the forward GAGTGGCCAACTCCATCAC and reverse 5′GTTATGTAACGCGGAACTCC3′ oligonucleotides to amplify AAV DNA. The pAAV plasmid was used as a positive control.

### Statistics

Statistical analysis was performed with Graph Pad Prism software (San Diego, CA). Results are expressed as means ± SEM. The significance of the difference between mean values was evaluated using one-way variance analysis (ANOVA) with a post hoc Bonferroni test in all experiments except that differences of mSEAP mRNA expression levels and activity in transduced cells with AAV and mutAAV were analysed using the unpaired Student’s t-test (two tailed) with a *p* threshold of 0.05.

## Electronic supplementary material

Supplementary Information
