# Supplementary material for: RFX1 and RFX3 Transcription Factors Interact with the D Sequence of Adeno-Associated Virus Inverted Terminal Repeat and Regulate AAV Transduction
Source: Sci Rep. 2018 Jan 9;8:210. doi: 10.1038/s41598-017-18604-3 (PMC5760533; doi:10.1038/s41598-017-18604-3)
Supplement: Supplementary file 1 — Supplementary Information [file 41598_2017_18604_MOESM1_ESM.pdf]

Supplementary Information

RFX1 and RFX3 Transcription Factors Interact with the D sequence of Adeno-Associated Virus Inverted Terminal Repeat and Regulate AAV Transduction

Laura Julien*[1], Julie Chassagne*[1], Cécile Peccate[1], Stéphanie Lorain[1], France Piétri-Rouxel[1], Olivier Danos[2] and Sofia Benkhelifa-Ziyyat[#1]

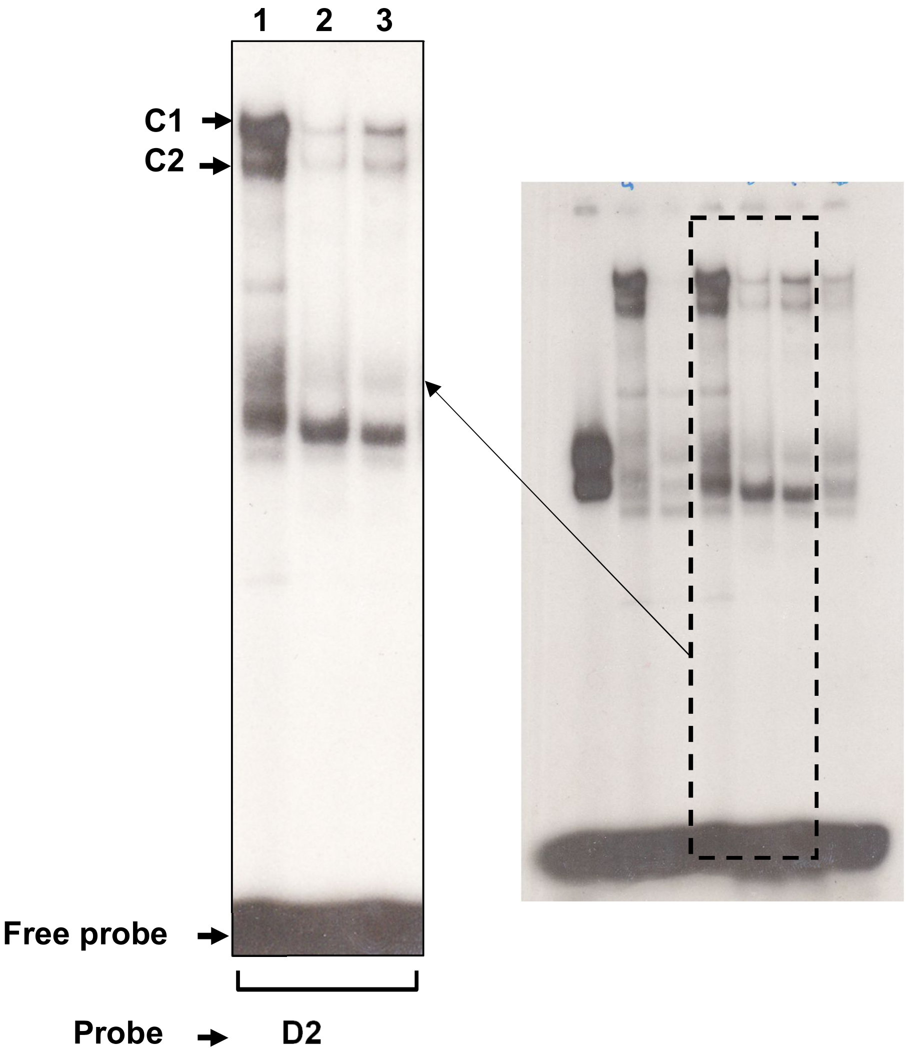

Supplementary figure S1: C1 and C2 complexes were also detected in nuclear extracts from HeLa and SUP-T1 cells. Lane 1: HEK-293, Lane 2: Supt1, Lane 3 : HeLa

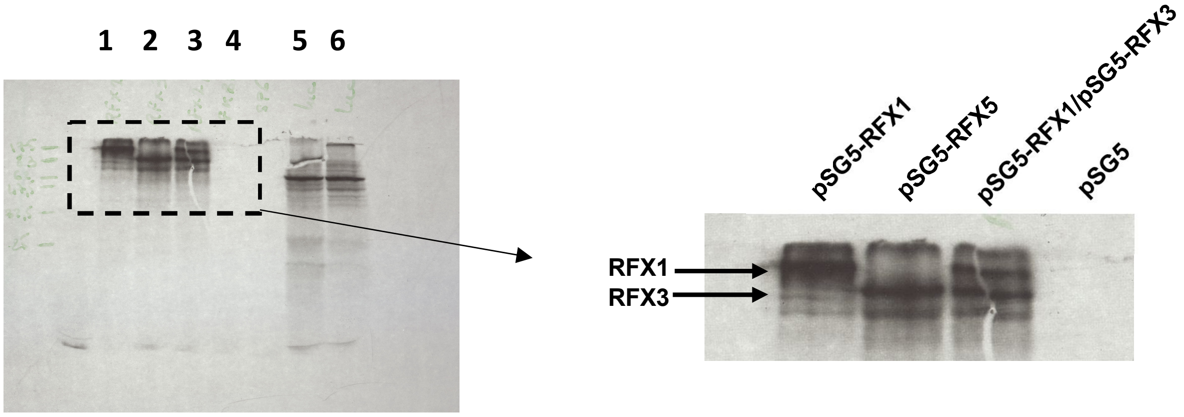

Supplementary figure S2: Human RFX1 and RFX3 cDNA subcloned in pSG5 vector were transcribed and translated in presence of 35S-methionine by T7 TNT coupled reticulocyte lysate system according to the manufacturer's protocol (Promega). Reaction products were resolved by SDS-polyacrylamide gel electrophoresis followed by exposure to a radiographic film. Lane 1: pSG5-RFX1, lane 2: pSG5-RFX3, lane 3: pSG5-RFX1 and pSG5-RFX3, lane 4: pSG5, lanes 5 and 6 pSG5-*Renilla* luciferase.

A

Batches	AAV-070	mutAAV-071	AAV-064	mutAAV-065	AAV-047	mutAAV-048
Titers vg/ml	3,20E+12	2,90E+12	1,10E+12	9,70E+11	1,30E+12	1,70E+12

Production 1   Production 2   Production 3

B

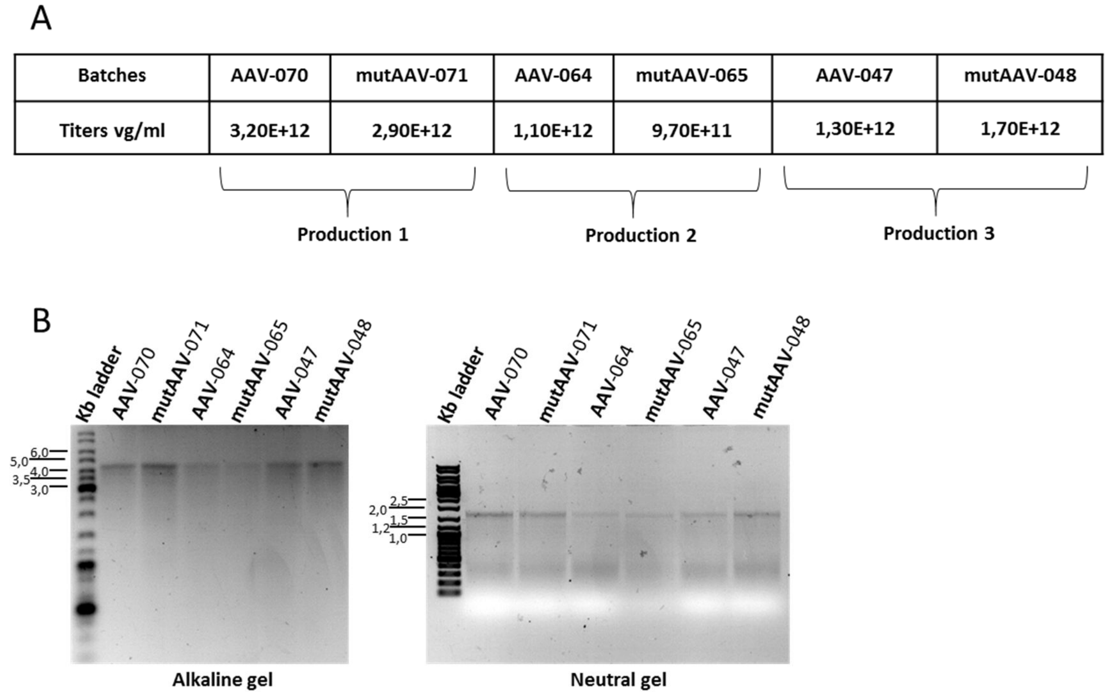

Supplementary figure S3: (**A**) **D2 mutation did not affect vector titers**. AAV-muSEAP (AAV-070, AAV-064 and AAV-047) and mutAAVmSEAP (mutAAV-071, mutAAV-065 and mutAAV-048) were produced and titered side by side (n=3: production 1, production 2 and production 3) as described in materials and methods section. (**B) Full length DNAs of mutAAV-mSEAP and AAV-mSEAP are equally packaged in particles.** The purified vector DNAs from the three different productions of either AAV-mSEAP or mutAAV-mSEAP used in this study were analyzed by alkaline and neutral agarose gel electrophoresis as described previously (Wang *et al.*, gene therapy, 2003). The single stranded AAV DNA of both AAVmSEAP and mutAAVmSEAP migrated in the alkaline agarose gel (denaturing-conditions) as a denatured single-stranded molecule of a 4.2 kilobase (Kb). As expected, in neutral agarose gel (non-denaturing conditions), the single stranded molecules of 4.2 Kb migrate faster next to the 2 kb dsDNA marker (undenatured).

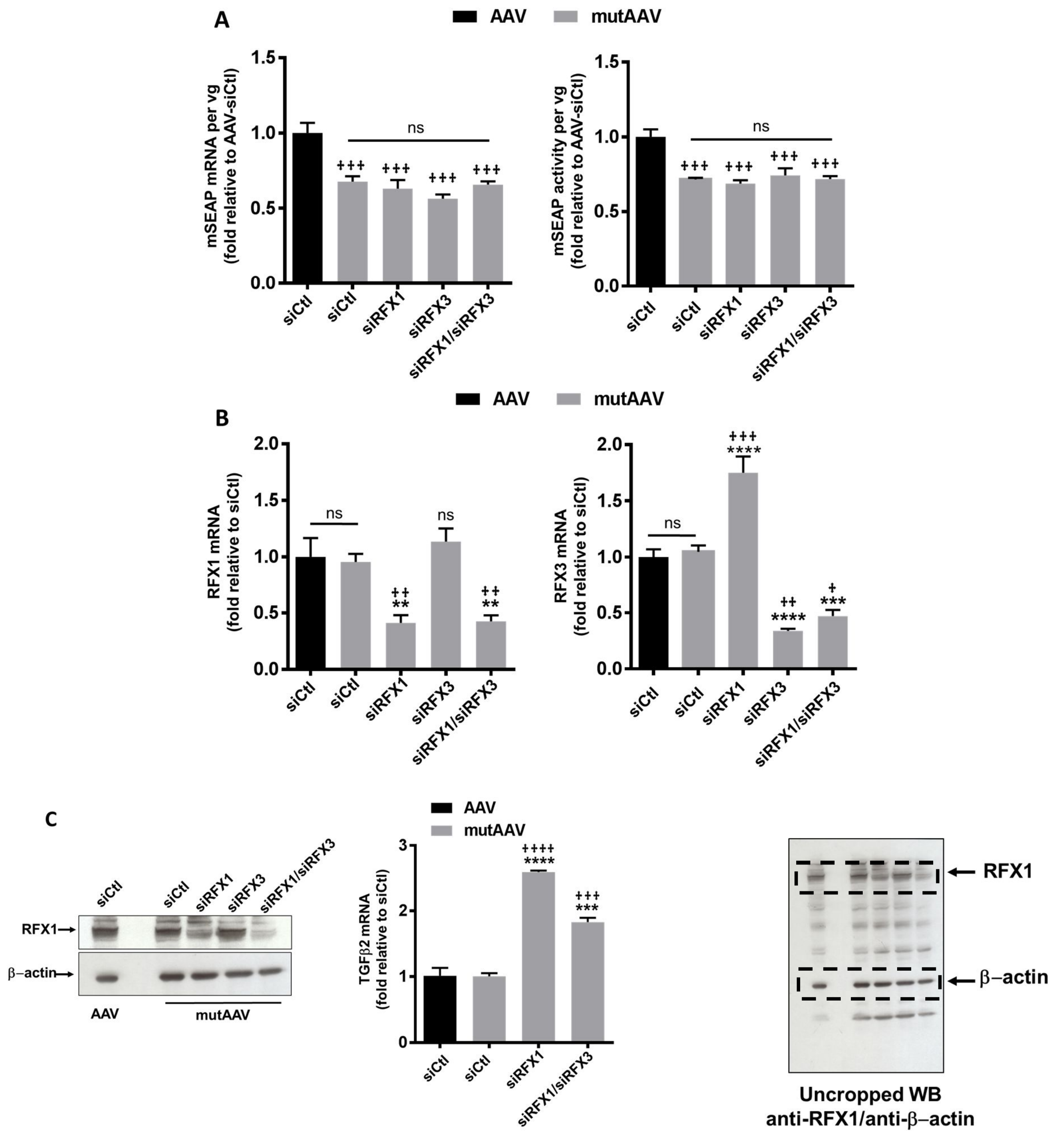

Supplementary figure S4: siRNA-mediated knockdown of RFX proteins did not impact mutAAV mediated transgene expression in HEK-293 cells. (A) Transduction efficiency of mutAAV-mSEAP (gray bars) in siCtl (control), siRFX1, siRFX3 and siRFX1/siRFX3 transfected cells compared to transduction efficiency of AAV-mSEAP in siCtl treated cells (Black bars). The AAV-mSEAP and mutAAV-mSEAP transduction efficiencies was assessed by quantifying the mSEAP mRNA expression level and activity 24h post-transduction. mSEAP activity and transcripts levels were normalized by the AAV vg copy number. **(B)** Real time RT-PCR analysis of RFX expression showing the efficacy of RFX siRNA silencing in HEK-293 cells transfected with siCtl, siRFX1, siRFX3 or both (siRFX1/RFX3). **(C)** siRNAs are functional in mutAAV transduced cells. **(Left panel)** RFX1 knock down in mutAAV transduced cells shown by western blotting led to (**Right panel**) the increase of TGFβ2 mRNA level as previously described in siRFX1 treated cells (Feng C and Zuo Z, J Biol Chem, 2012). The data are represented as the mean ± SEM of two independent experiments performed in quadruplicates. Data that were statistically different from mutAAV-siCtl and from AAV-siCtl are marked respectively *p<0.05; **p<0.01, ***p<0.001, ****p<0.0001 and $^{+}$p<0.05; $^{++}$p<0.01, $^{+++}$p<0.001, $^{++++}$p<0.0001. ns, non-significant.

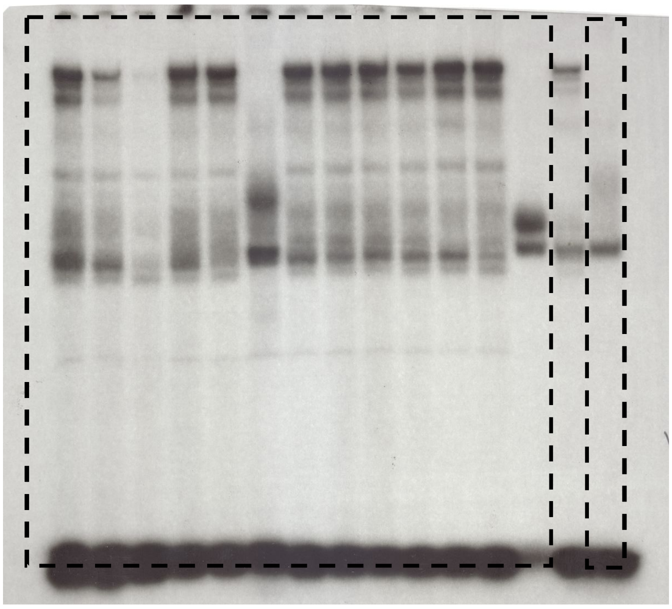

Supplementary figure S5: Uncropped image of gel presented in figure 1

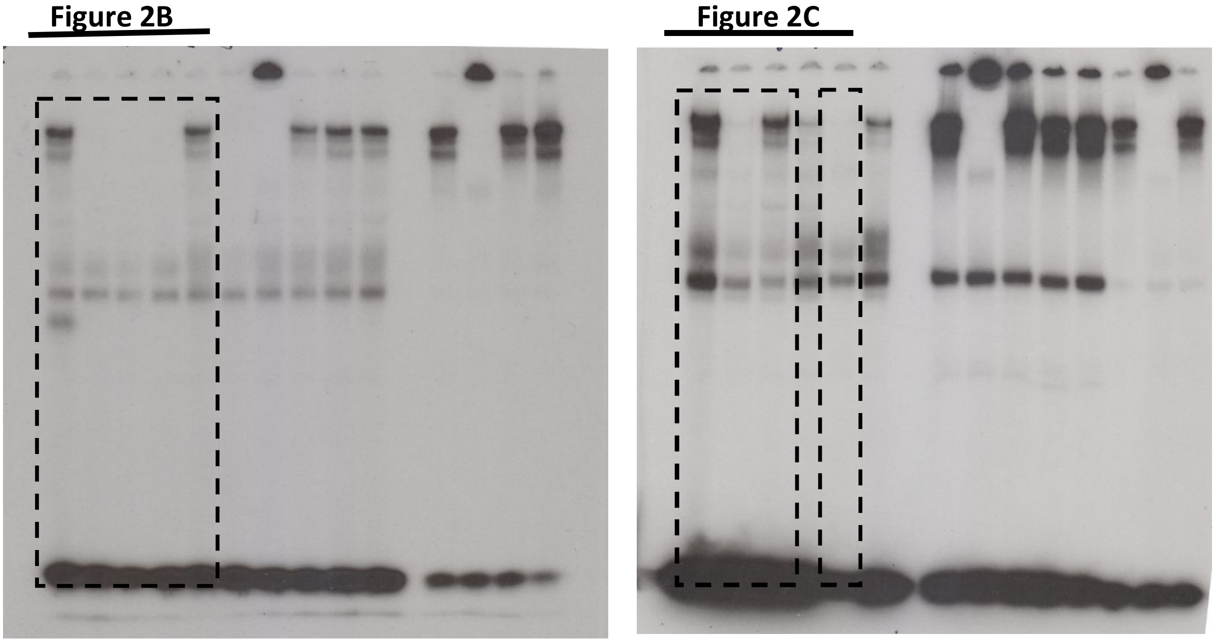

Supplementary figure S6: Uncropped image of gels presented in figure 2

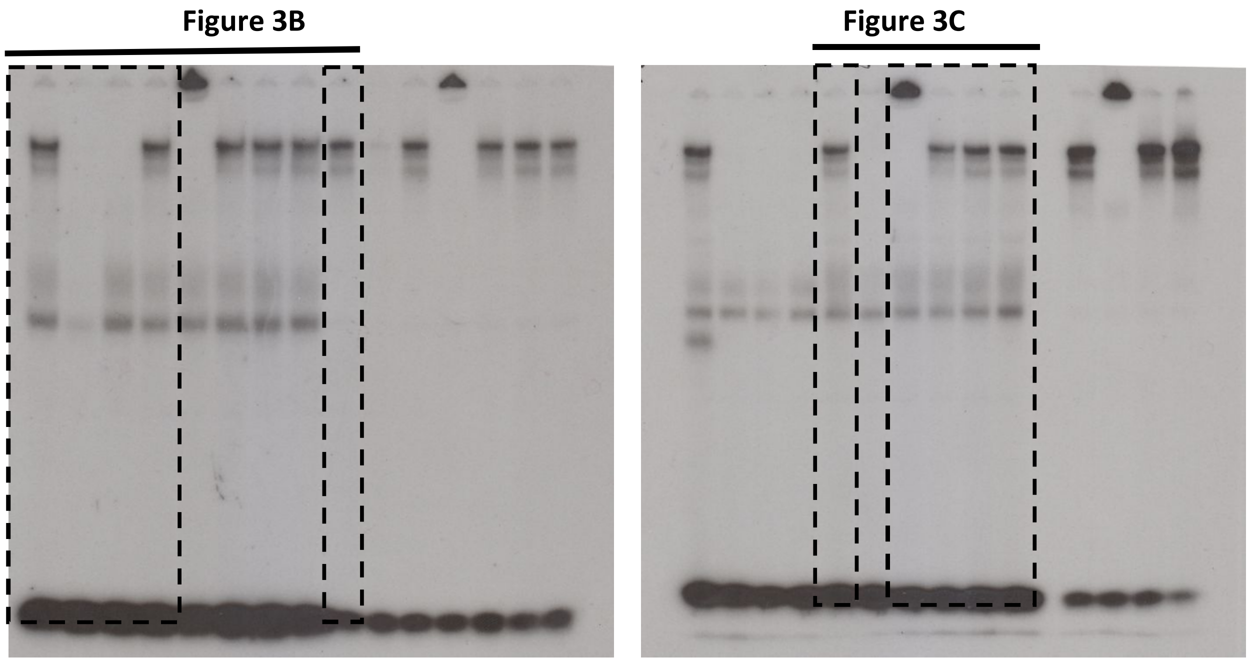

Supplementary figure S7: Uncropped image of gels presented in figure 3

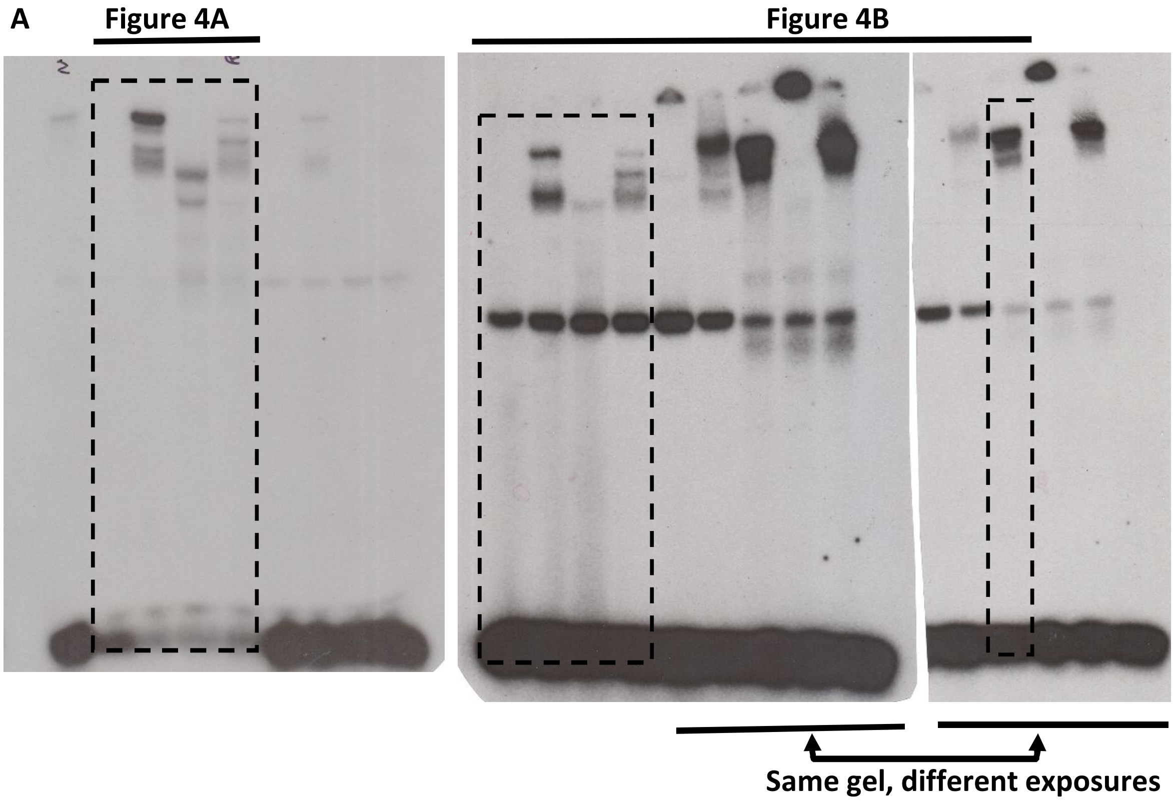

Supplementary figure S8: (**A**) Uncropped image of gels presented in figure 4.

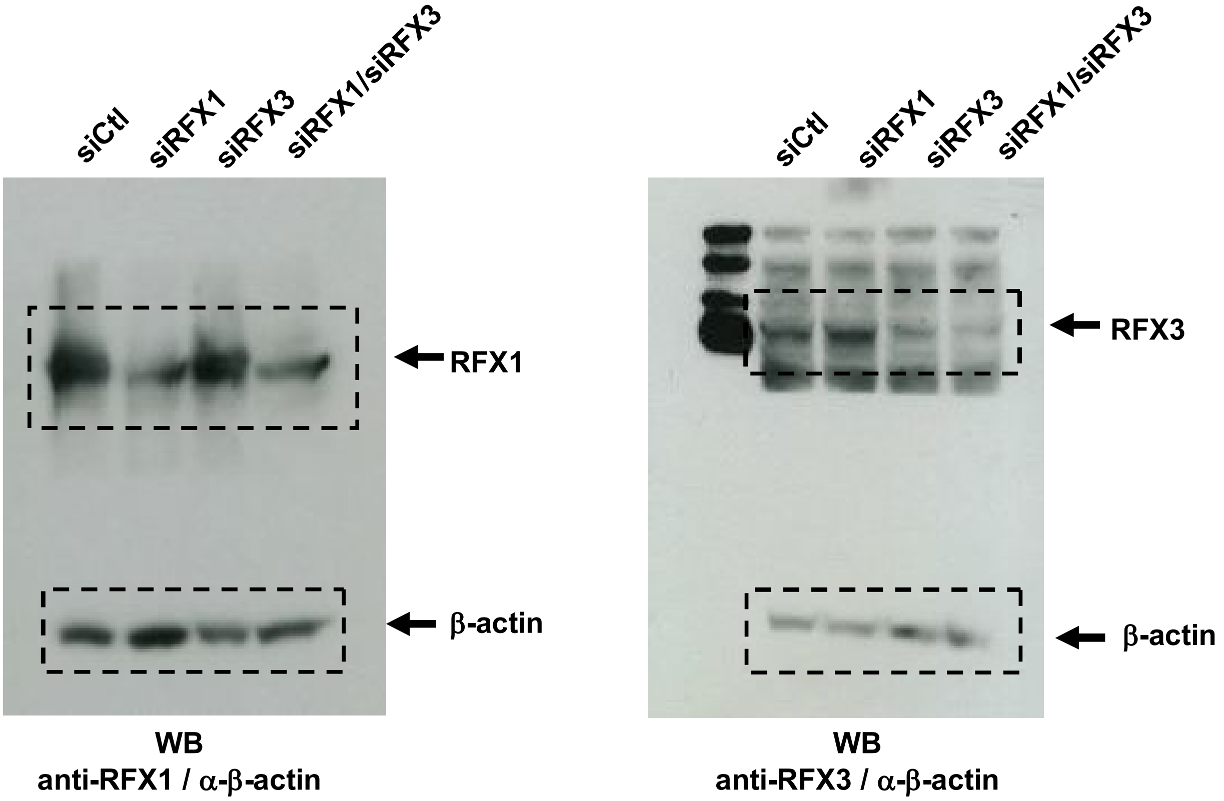

Supplementary figure S9: Uncropped image of the western blot presented in figure 5

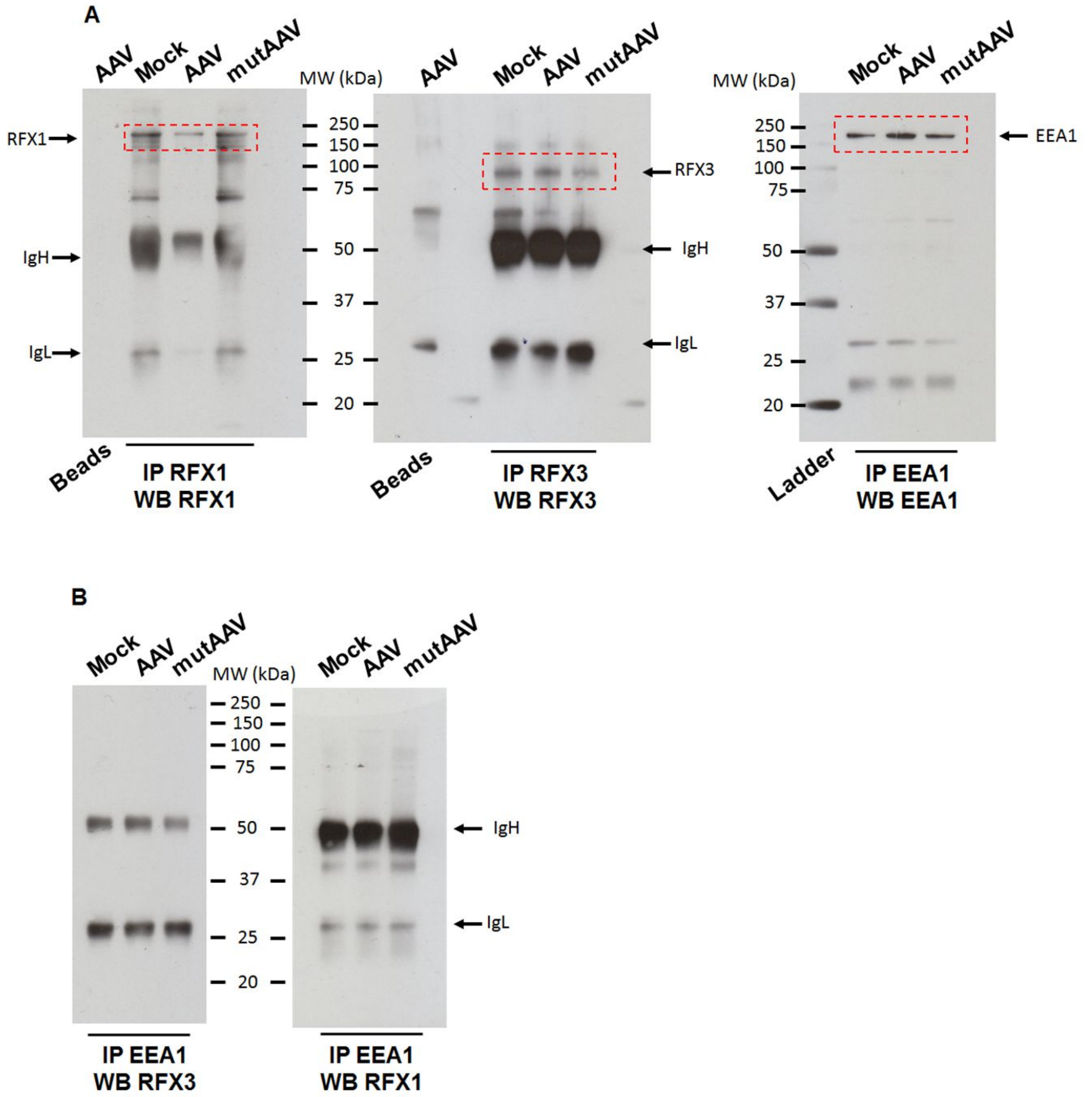

Supplementary figure S10: **(A)** Uncropped image of gels presented in figure 6B. **(B)** Early Endosome Antigen 1 (EEA1) antibody does not precipitate RFX proteins.
IP: Immunoprecipitation, WB: Western Blott
